# Caesarean delivery and subsequent pregnancy interval: a systematic review and meta-analysis

**DOI:** 10.1186/1471-2393-13-165

**Published:** 2013-08-27

**Authors:** Sinéad M O’Neill, Patricia M Kearney, Louise C Kenny, Tine B Henriksen, Jennifer E Lutomski, Richard A Greene, Ali S Khashan

**Affiliations:** 1 National Perinatal Epidemiology Centre, Department of Obstetrics and Gynaecology, 5th Floor, Cork University Maternity Hospital, Wilton, Cork, Ireland; 2 Department of Epidemiology and Public Health, University College Cork, Cork, Ireland; 3 The Irish Centre for Fetal and Neonatal Translational Research (INFANT), University College Cork, Cork, Ireland; 4 Perinatal Epidemiology Research Unit, Department of Paediatrics, Aarhus University Hospital, Skejby, Aarhus, Denmark

## Abstract

**Background:**

Caesarean delivery has increased worldwide, however, the effects on fertility are largely unknown. This systematic review aims to compare subsequent sub-fertility (time to next pregnancy or birth) among women with a Caesarean delivery to women with a vaginal delivery.

**Methods:**

Systematic review of the literature including seven databases: CINAHL; the Cochrane Library; Embase; Medline; PubMed; SCOPUS and Web of Knowledge (1945 - October 2012), using detailed search-strategies and reference list cross-checking. Cohort, case–control and cross-sectional studies were included. Two assessors reviewed titles, abstracts, and full articles using standardised data abstraction forms and assessed study quality.

**Results:**

11 articles were eligible for inclusion in the systematic review, of these five articles which adjusted for confounders were combined in a meta-analysis, totalling 750,407 women using fixed-effect models. Previous Caesarean delivery was associated with an increased risk of sub-fertility [pooled odds ratio (OR) 0.90; 95% CI 0.86, 0.93]. Subgroup analyses by parity [primiparous women: OR 0.91; 95% CI 0.87, 0.96; not limited to primiparous women: OR 0.81; 95% CI 0.73, 0.90]; by publication date (pre-2000: OR 0.80, 95% CI 0.68, 0.94; post-2000: OR 0.90, 95% CI 0.86, 0.94); by length of follow-up (<10 years: OR 0.81, 95% CI 0.73, 0.90; >10 years: OR 0.91, 95% CI 0.87, 0.96); by indication for mode of delivery (specified: 0.92, 95% CI 0.88, 0.97; not specified: OR 0.81, 95% CI 0.73, 0.90); by cohort size (<35,000: OR 0.79, 95% CI 0.67, 0.92; >35,000: OR 0.90, 95% CI 0.87, 0.95), by definition of sub-fertility used divided into (birth interval [BI]: OR 0.89, 95% CI 0.84, 0.94; inter-pregnancy interval [IPI]: OR 0.91, 95% CI 0.85, 0.97; and categorical measures: OR 0.81, 95% CI 0.73, 0.90); continuous measures: OR 0.91, 95% CI 0.87, 0.96) were performed. Results of the six studies not included in the meta-analysis (which did not adjust for confounders) are presented individually.

**Conclusions:**

The meta-analysis shows an increased waiting time to next pregnancy and risk of sub-fertility among women with a previous Caesarean delivery. However, included studies are limited by poor epidemiological methods such as variations in the definition of time to next pregnancy, lack of confounding adjustment, or details of the indication for Caesarean delivery. Further research of a more robust methodological quality to better explore any underlying causes of sub-fertility and maternal intent to delay childbearing is warranted.

## Background

Over the past three decades, rates of Caesarean delivery have increased dramatically worldwide [1,2]. In the United Kingdom (UK) for example, 2% of all births were delivered via Caesarean section in 1953, 18% in 1997 and 21% in 2001 [3]. In 2010, the Caesarean delivery rate was 24.8% in the UK [4]. Statistics from 2010–2011 for Australia and the United States of America (USA) show almost one in every three pregnant women has a Caesarean delivery [5,6].

A number of possible biological mechanisms for an association between Caesarean delivery and sub-fertility have been postulated including infection at the site of the wound, scar adhesion and placental bed disruption [7]. The waiting time to next pregnancy or birth is reported to be a robust surrogate marker of sub-fertility in the literature [8]. A number of studies to date have assessed the long-term consequences of Caesarean delivery on subsequent fertility [7,9,10] including two reviews [11,12]. To date, few studies have found that women with a Caesarean delivery are less likely to have a subsequent pregnancy and have a longer pregnancy interval compared to women with a vaginal delivery, even after adjustment for parity [9,11,13-15]. In addition, one study examined a sub-group of women with pre-eclampsia and it was reported that women who delivered by Caesarean were 20% less likely to have another pregnancy [16]. Other studies have claimed a Caesarean delivery does not affect future fertility [17,18].

A recent systematic review and meta-analysis investigating the rate of continuation to a subsequent pregnancy reported that women with a previous Caesarean delivery are 9% less likely to have a subsequent pregnancy and 11% less likely to have a subsequent birth [12]. This systematic review however did not meta-analyse subsequent pregnancy interval, which is arguably a more robust marker of sub-fertility. However, it must be noted that women who have a Caesarean delivery may differ in many respects, for example, in height, age, body mass index (BMI) and obstetric history. Thus, any association between mode of delivery and subsequent sub-fertility may be confounded by a variety of medical, obstetrical, social and socioeconomic confounding factors [19-23]. Furthermore, residual confounding such as the indication for Caesarean delivery may explain part or all of the association between Caesarean delivery and sub-fertility [18].

There are an estimated 70 million couples worldwide who are infertile and the long-term clinical outcomes associated with sub-fertility are uncertain [24]. With one in every six couples reported to be seeking fertility treatment [25] any potential association between mode of delivery and subsequent sub-fertility could have major social, clinical and public health implications.

Therefore, the purpose of this systematic review and meta-analysis is to update, compile and critically review the existing evidence on the effects of a Caesarean delivery on the waiting time to subsequent pregnancy or birth and to provide a quantitative estimate of the overall relationship between mode of delivery and subsequent sub-fertility.

## Methods

### Primary objective

The main objective of this systematic review and meta-analysis is to collect and interpret the published literature on the association between a Caesarean delivery and subsequent sub-fertility (time to next pregnancy or birth) and to calculate a pooled estimate of the odds of sub-fertility following a Caesarean delivery.

### Primary outcome

The outcome of interest is subsequent sub-fertility, defined as pregnancy interval (the waiting time to next pregnancy or birth). Pregnancy interval has been classified according to the standard definitions used in the literature to date for the purpose of this systematic review and meta-analysis as follows:

1. *Inter-pregnancy interval* (IPI), defined as the time passed since the termination of the previous pregnancy and the conception of the next pregnancy, or

2. *Birth interval (BI)* defined as the time elapsed from the date of delivery of the previous child to the date of delivery of the subsequent child.

The outcome will be a dichotomous measure of the time to next pregnancy within the follow-up time period reported in each study. Where a study reports estimates of two separate intervals as a measure of sub-fertility (i.e. a period of >1 year or a period of >3 years trying to conceive), the longer time interval will be used in the meta-analysis. Furthermore, where a study provides stratified estimates and sub-group analyses, only the overall estimate of sub-fertility will be included in the meta-analysis.

### Search strategy

The protocol for this systematic review and meta-analysis was registered on PROSPERO, the international prospective register of systematic reviews (unique identification number: CRD42012003166) and is available in full on the National Institute for Health Research (NIHR) website [26]. This systematic review and meta-analysis was conducted in accordance with the preferred reporting items for systematic reviews and meta-analyses statement (PRISMA) [27], which is a detailed checklist of items specifically designed for this purpose. We performed a comprehensive search of the published literature using a combination of medical subject headings (MeSH) or key word terms for Caesarean delivery and sub-fertility (time to next pregnancy including IPI or BI). We searched the following databases for potentially eligible studies: CINAHL (1981 to October 17^th^ 2012), the Cochrane Library (1993 to October 17^th^ 2012), Embase (1974 to October 17^th^ 2012), Medline (1966 to October 17^th^ 2012), PubMed (1966 to October 17^th^ 2012), SCOPUS (1960 to October 17^th^ 2012) and Web of Knowledge (1945 to October 17^th^ 2012). Terms for Caesarean delivery included *Caesarean section, Caesarean delivery, abdominal delivery, C-section, mode of delivery,* and *post Caesarean delivery*. Terms for pregnancy interval included *birth interval, birth spacing, pregnancy interval, first birth interval, inter-delivery interval, pregnancy gap* and *pregnancy spacing* (Additional file 1). We enhanced our electronic search by cross-checking the reference lists of all relevant studies. We included studies which examined the association between mode of delivery and sub-fertility defined as time to next pregnancy or birth. No date or language restrictions were imposed. Eligibility criteria for inclusion in the systematic review included:

1. Data were from an original study (i.e. no review articles, editorials or commentaries).

2. Study design: cohort, cross-sectional or case–control studies, in which mode of delivery and the subsequent IPI or BI were reported.

3. Outcome definitions: studies which defined sub-fertility using the standard pregnancy interval definitions (IPI or BI) were considered appropriate for inclusion in the systematic review and meta-analysis.

Eligibility criteria for inclusion in the meta-analysis (in addition to the above criteria) included:

4. Reporting of an adjusted effect estimate (relative risk [RR], odds ratio [OR], or hazard ratio [HR]) or sufficient data in order to calculate these estimates of the relationship between Caesarean delivery and subsequent sub-fertility.

### Study selection and data abstraction

Titles and abstracts of studies retrieved from the search strategy were reviewed independently applying the appropriate inclusion and exclusion criteria by the researchers. The full text article was obtained for all potentially eligible studies for further appraisal. Using a standardised data abstraction form, two assessors (SMON, ASK) individually entered the following data from each study: first author’s name, year, study design, location of the study (country), study period and follow-up, data source (i.e. hospital database, nationwide registers, patient records), total sample size, measure of pregnancy interval used (IPI or BI, and whether it was measured as a continuous or categorical outcome), statistical tests used, the indication for Caesarean delivery (breech, elective, emergency), the population sampled (i.e. primiparous, multiparous women), the exclusion criteria and the main results and conclusions of each study. Discrepancies in data abstraction between assessors were resolved through consensus.

### Statistical analysis

#### Primary analysis

The primary analysis estimated the relationship between prior Caesarean delivery and subsequent sub-fertility (pregnancy interval between the index and subsequent pregnancy) compared to women with a prior vaginal delivery. The generic inverse variance method with a fixed-effect model was used to calculate pooled estimates across the studies. A funnel plot was produced to estimate the likelihood of publication bias using the pooled OR and standard error (SE). Only studies which reported adjusting for a minimum of three confounders including maternal age were included in the meta-analysis. Studies which reported crude estimates are presented individually in a separate table.

#### Subgroup analyses

Firstly, we estimated separate ORs by parity (primiparous versus not limited to primiparous women), to assess the degree of confounding by number of previous deliveries. Secondly, we estimated the pooled OR by publication date (pre-2000, post 2000). Thirdly, we performed an analysis by length of follow-up (<10 years, >10 years). Next we performed a separate analysis based on whether an indication for mode of delivery was available or not (yes, not specified). Next we carried out an analysis by cohort size (<35,000, >35,000) and finally by definition of sub-fertility used: (a) BI versus IPI and (b) categorical measures (e.g. <1 year, >3 years) vs. continuous measures

Analyses were performed using Review Manager version 5.1 software [28].

### Heterogeneity assessment

Heterogeneity between the included studies was assessed by examining the differences in study characteristics including study setting (high-income developed country versus low-income developing country), study design (case–control, cohort or cross-sectional), sampling frame (population-based or single/select institutions), and definition of the outcome measure used. In the meta-analysis, we used the I^2^ statistic to ascertain statistical heterogeneity, following the Cochrane Handbook for Systematic Reviews threshold recommendations [29]. An I^2^ statistic value between 0% and 40% suggests heterogeneity may not be important; 30% to 60% represents moderate heterogeneity; 50% to 90% represents substantial heterogeneity; and 75% to 100% represents considerably significant heterogeneity. The magnitude and direction of the effects (Chi-squared test P-value, 95% confidence interval for I^2^) determine the importance of the I^2^ statistic according to the handbook.

### Quality assessment

The methodological quality was assessed using a quality assessment tool based on six different types of bias common in observational studies (selection, exposure, outcome, analytic, attrition and confounding) for each study included in the review. This bias classification tool has been described in detail elsewhere [30]. Study bias was recorded as minimal, low, moderate or high according to the perceived degree of bias present for each of the six types of bias. For example, for confounding factor bias a study would be classified as minimal in bias if it adjusted for at least three key confounders including maternal age. An overall likelihood of bias was then estimated based on the total amount of bias perceived to be present.

Ethical approval was not required for this systematic review and meta-analysis as it did not include any experimental research on humans or personal information which would otherwise require informed consent.

## Results

### Database search results

**Figure 1:**
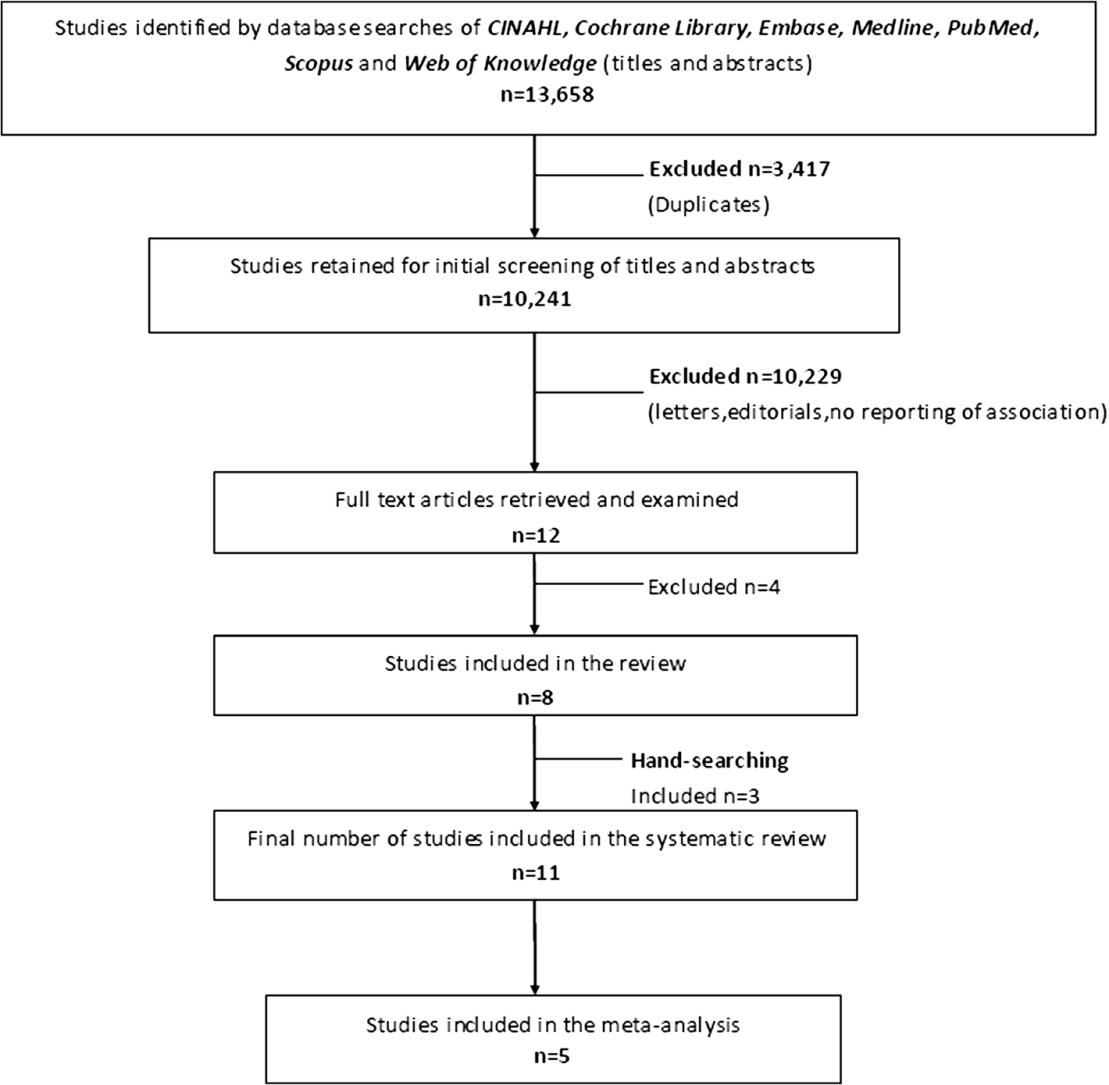
**Study selection.** Flow chart of identification and selection of studies for inclusion in the systematic review.

The database searches produced 13,658 citations, of which the titles and abstracts of 10,241 were screened after removal of 3,417 duplicates. Of these, 12 were considered potentially relevant and the full text was obtained (Figure 1). From here, four were excluded with the main reasons being lack of data on the exposure (mode of delivery) or the outcome of interest (sub-fertility defined as time to next pregnancy/birth) and one study [31] used the same data source as another eligible study [16] and so in order to avoid duplication of data was removed. After hand-searching the reference lists of eligible studies, a further three studies were obtained yielding a total of 11 studies which met the inclusion criteria of the systematic review.

#### Characteristics of studies included in the review

The characteristics of the included studies are presented in Table 1. Five studies defined time to next pregnancy using IPI [7,10,18,32,33], five studies used BI [16,34-37] and one study reported both [17]. Six studies [16-18,32,33,36] reported time to next pregnancy on a continuous scale (e.g. median IPI or BI in days), whilst five studies [7,10,34,35,37] categorised time to next pregnancy (e.g. >3 years trying to conceive or conceiving within five years). Four studies were published in the 1980’s [33,35-37], one study in the 1990’s [34] and six from the year 2000 on [7,10,16-18,32]. Study populations ranged in size from 547 to 596,341 women [16,32] and were conducted in countries including the Netherlands [32], Scotland [18], Norway [16], England [7,17], Brazil [34], Sweden [37] and the USA [33,35,36]. One study included 22 sub-Saharan African countries [10]. Length of follow-up ranged from a minimum of one year [36] to a maximum of 34 years [16].

**Table 1:** Characteristics of studies included in the systematic review

**Study, year**	**Country/ Study design**	**Study period/ Follow-up**	**Data source**	**Total sample**	**Interval used**	**Statistical tests used**	**Indication for Caesarean**	**Parity**	**Exclusions**
Eijsink, 2008 [32]	Holland / Cohort	1998-2002/ Follow-up to 2005	Single hospital database	547	IPI (scale)	Kaplan-Meier curves	Breech emergency & elective	Primiparous	Multiple deliveries, congenital malformations, uterine anomalies
Collin, 2006 [10]	Sub-Saharan Africa / Cohort	1993-2003/ Minimum follow-up 3 years	Cross-sectional standardised survey data from 22 countries	35,398	IPI ( >1 or <5 years)	Cox regression	No	Multiparous	Women with >1 terminated pregnancy; women currently breastfeeding; abstaining from intercourse or using contraception; women in South Africa (due to high elective Caesarean rates); stillbirths; women outside 14–49 years of age
Smith, 2006 [18]	Scotland / Cohort	1980-1984/ Follow-up to 1999	Population-based database	109,991	IPI (scale)	Logistic regression	Emergency, elective for breech, all other pre-labour Caesarean	Primiparous	Multiple births, preterm births, perinatal deaths, missing values
Murphy, 2002 [7]	UK / Cohort	1991-1992/ Minimum follow-up 3 years	Self-completed questionnaire data	3,994	IPI (>1 or >3 years)	Logistic regression	No	Multiparous	Women with unplanned pregnancies
Zdeb, 1984 [33]	USA/ Cohort	1975/ Follow-up for 5 years	Single hospital database	5,513	IPI (scale)	X^2^ analysis	No	Primiparous	Stillbirths, high-forceps, mid-forceps, breech, version and extraction deliveries
Tollånes, 2007 [16]	Norway/ Cohort	1969-1996/ Follow-up to 2003	Nationwide Birth Register	596,341	BI (scale)	Logistic regression	Breech, pre-eclampsia, low-risk Caesarean groups	Primiparous	Women dying aged <50, women who changed partners, multiple pregnancies
Tower, 2000 [17]	UK / Cohort	1992-1993/ Follow-up for 5 years	Single hospital database	1,152	BI, IPI (scale)	Not stated	Failure to progress, fetal distress	Primiparous	None stated
Huttly, 1990 [34]	Brazil/ Cohort	1982/ Follow-up for 4 years	Data obtained from interview of mothers	4,683	BI (within 35–52 months)	Not stated	No	Multiparous	Tubal ligation/ sterilisation
Hemminki,1987 [37]	Sweden/ Cohort	1973, 1976/ Follow-up for 5 years	Nationwide Birth Register survey data	12,918	BI (<5 years)	X^2^ analysis	No	Primiparous	Hysterectomy, non-Swedish women, rare blood groups, multiple pregnancies, malformations, birth weight <2000g, perinatal death
LaSala, 1987 [35]	USA/ Case control	1978/ Follow-up for 3 years	Single hospital logbook	570	BI (>2 years)	X^2^ analysis	No	Primiparous	Missing hospital records, sterilisation, women using contraception
Hemminki,1985 [36]	USA/ Cohort	1957-1982/ Minimum 1 year follow-up, maximum 25 years	Nationwide Birth Register	812	BI (scale)	Kaplan-Meier curves	No	Primiparous	Women outside 15–44 years of age; women living in Alaska or Hawaii; women with no live births or more than two abortions; multiple births; missing data; perinatal deaths; birth weight <1500g; women who put their child up for adoption

**Table legend: *IPI*** Inter-pregnancy interval, ***BI*** Birth interval. Data for interval categories: **Continuous** refers to the median/mean IPI or BI, **>1 year** refers to trying to conceive for more than 1 year, **>3 years** refers to trying to conceive for greater than 3 years, **<5 years** refers to conceiving within 5 years, **>2 years** refers to being sub-fertile for greater than 2 years.

Three studies used national registers [16,18,37], three used smaller databases (i.e. a single hospital obstetric database) [17,32,33], four studies used interview or questionnaire survey data [7,10,34,36], and one study used patient medical records [35]. Eight studies included primiparous women only (i.e. those women with only one previous pregnancy) [16-18,32,33,35-37] and three were not limited to primiparous women [7,10,34]. Studies included 10 cohort [7,10,16-18,32-34,36,37], and one case–control [35]. Indication for mode of delivery was reported in four studies [16-18,32]. Exclusion criteria, confounder adjustment and matching techniques varied between studies and only one study was conducted in a low-income region (Sub-Saharan Africa) [10].

#### Primary outcome analysis

**Figure 2:**
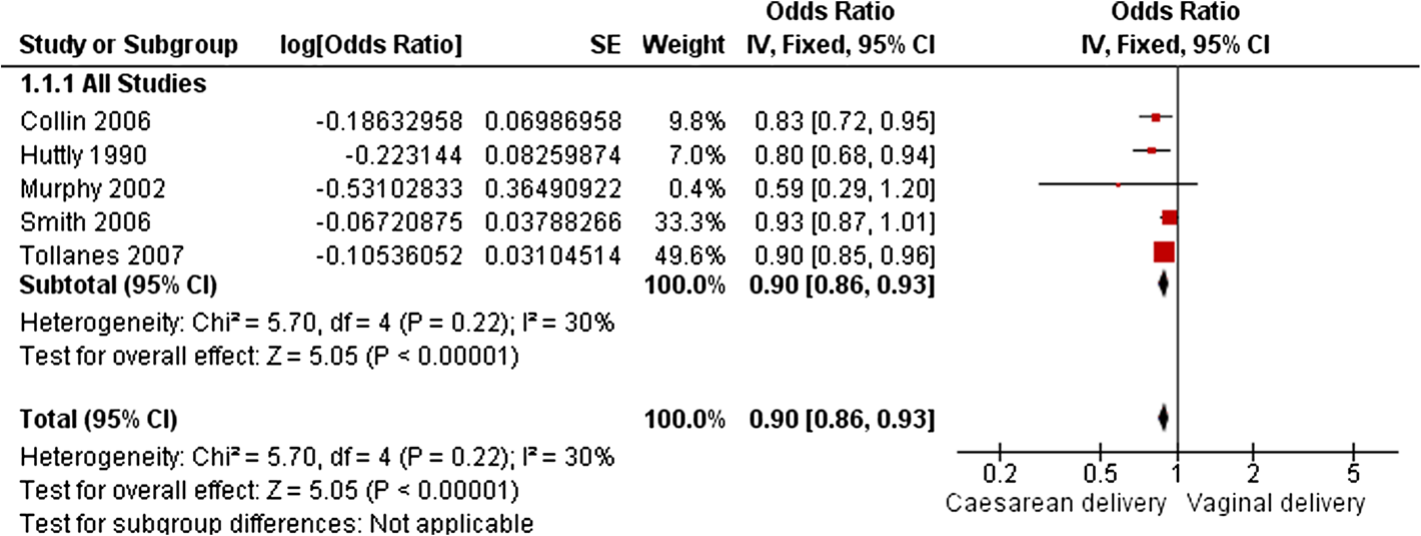
**Caesarean section and subsequent sub-fertility.** Fixed-effect model of the relationship between Caesarean delivery and subsequent sub-fertility (time to next pregnancy or birth) compared to vaginal delivery from five published studies including 750,407 women.

**Figure 3:**
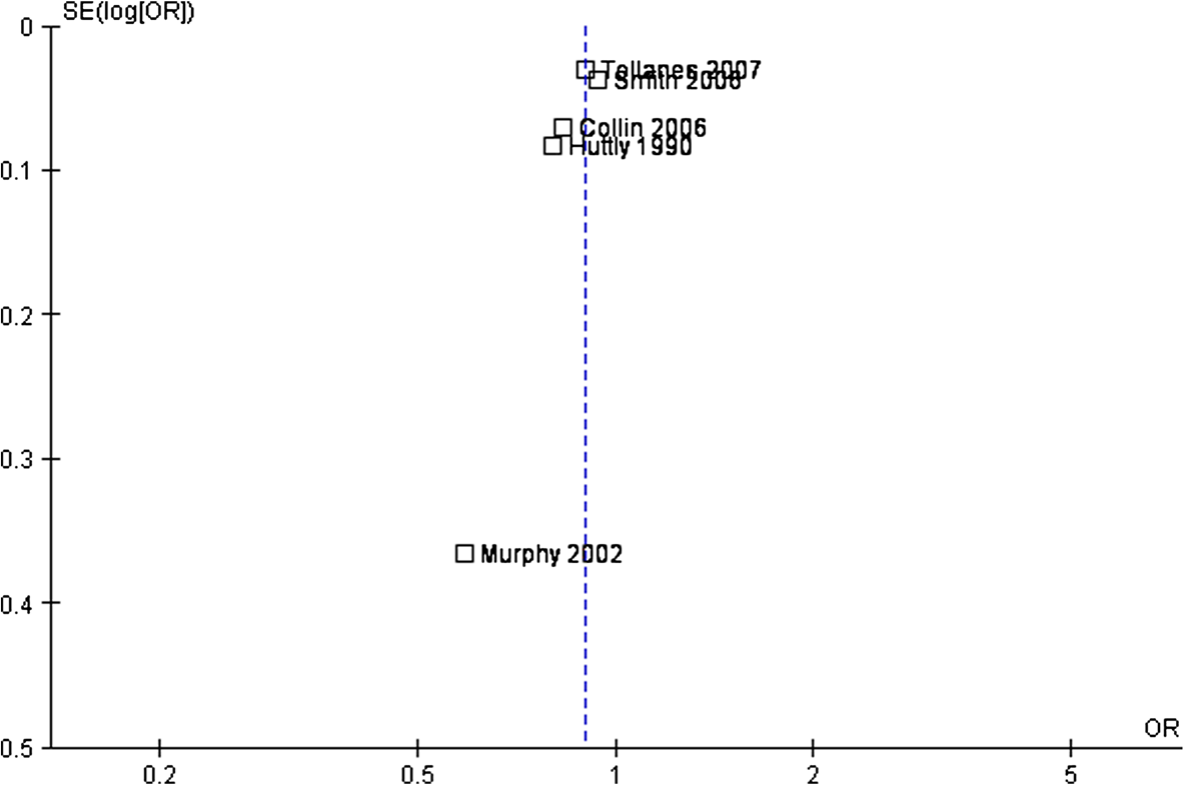
**Funnel plot.** Funnel plot assessing publication bias in the relationship between Caesarean delivery and subsequent sub-fertility (time to next pregnancy or birth) compared to vaginal delivery from five published studies.

Five studies adjusted for confounders and are included in the meta-analysis with data on 750,407 women available. A fixed-effect model is reported as heterogeneity between the studies was considered low to moderate (I^2^ = 30%, P = <0.0001). The pooled adjusted OR of sub-fertility is 0.90 (95% CI 0.86, 0.93) (Figure 2). Inspection of the funnel plot (Figure 3) showed that one study [7] may attribute some degree of publication bias, although, there are other possible explanations.

#### Subgroup analyses

Subgroup analyses are presented in Table 2. An OR of 0.91 (95% CI 0.87, 0.96) was generated for primiparous women and an OR of 0.81 (95% CI 0.73, 0.90) for studies not limited to primiparous women. For publication date, studies published pre-2000 yielded an OR of 0.80 (95% CI 0.68, 0.94), whilst studies published post-2000 revealed an OR of 0.90 (95% CI 0.86, 0.94). Length of follow-up <10 years produced an OR of 0.81 (95% CI 0.73, 0.90) compared to an OR of 0.91 (95% CI 0.87, 0.96) for studies with a follow-up period of >10 years. Studies which reported the indication for mode of delivery (including breech Caesarean delivery, elective Caesarean delivery, and emergency Caesarean delivery, etc.) yielded an OR of 0.92 (95% CI 0.88, 0.97) compared to an OR of 0.81 (95% CI 0.73, 0.90) for studies which did not report an indication for mode of delivery. Cohorts including a sample of <35,000 women yielded an OR of 0.79 (95% CI 0.67, 0.92) compared to cohorts >35,000, (OR 0.90, 95% CI 0.87, 0.95). Studies reporting sub-fertility using BI reported an OR of 0.89 (95% CI 0.84, 0.94) compared to an OR of 0.91 (95% CI 0.85, 0.97) for studies using IPI as the definition of sub-fertility. Studies using a categorical measure of sub-fertility reported an OR of 0.81 (95% CI 0.73, 0.90) compared to studies using a continuous measure (OR 0.91, 95% CI 0.87, 0.96).

**Table 2:** Subgroup analyses of the impact of Caesarean section on subsequent sub-fertility (time to next pregnancy or birth)

**Study characteristic**	**Number of studies included**	**Pooled OR estimate (95% CI)**	**I**^**2 **^**%**
***Overall pooled estimate***	n = 5	0.90 [0.86, 0.93]	30%
***Parity***			
Primiparous women only	n = 2	0.91 [0.87, 0.96]	0%
Not limited to primiparous women	n = 3	0.81 [0.73, 0.90]	0%
***By publication date***			
Pre-2000	n = 1	0.80 [0.68, 0.94]	NA
Post-2000	n = 4	0.90 [0.86, 0.94]	19%
***Length of follow-up***			
<10 years	n = 3	0.81 [0.73, 0.90]	0%
>10 years	n = 2	0.91 [0.87, 0.96]	0%
***Indication for mode of delivery***			
Specified*	n = 2	0.92 [0.88, 0.97]	88%
Not specified	n = 3	0.81 [0.73, 0.90]	0%
***By cohort size***			
<35,000	n = 2	0.79 [0.67, 0.92]	0%
>35,000	n = 3	0.90 [0.87, 0.95]	13%
***By definition of sub-fertility used***			
a. Birth Interval (BI)	n = 2	0.89 [0.84, 0.94]	44%
Inter-pregnancy interval (IPI)	n = 3	0.91 [0.85, 0.97]	45%
b. Categorical measure (e.g. > 3 years)	n = 3	0.81 [0.73, 0.90]	0%
Continuous measure (e.g. median IPI)	n = 2	0.91 [0.87, 0.96]	0%

**Table Legend:** Data refer to the pooled odds ratio (OR) and 95% confidence interval (CI) for each subgroup analysis. **I**^**2**^ is a statistical measure of heterogeneity between 0% and 100%, with higher percentages implying great heterogeneity. *Specified: refers to the indication for mode of delivery and includes in Smith (2006) [18] assisted (instrumental) vaginal delivery, vaginal breech, elective Caesarean for breech, emergency Caesarean and all other prelabour Caesareans. For Tollanes (2007) [16] includes: breech presentation, low risk obstetric group, and a pre-eclampsia group.

#### Results of studies included in the systematic review (but not the meta-analysis)

##### Inter-pregnancy interval (IPI)

Six studies were not eligible for inclusion in the meta-analysis as they did not report adjusted estimates. The results of these studies are presented in Table 3. Of the studies that used IPI to measure time to next pregnancy, one reported a longer IPI following Caesarean delivery (elective Caesarean delivery 22 months versus vaginal delivery 16 months; a difference of six months) [32]. Equally however, one study [33] reported that a Caesarean delivery was not associated with any delay in subsequent IPI. Median IPI in the overall cohort was reported as 21.8 months.

**Table 3:** Main findings of studies included in the systematic review

**Study**	**Main Findings**	**Conclusions**
Eijsink et al., 2008 [32]	**Median IPI:** Elective CS breech (22mths), Emergency CS (18 mths), Vaginal breech (16 mths), Reference [home birth] (18 mths)	In women with a breech delivery, a longer IPI among the elective CS group was reported, however there were only 35 women in this group
Zdeb et al., 1984 [33]	**Median IPI in mths:** 21.8	No significant difference in the timing of subsequent pregnancies among the two groups
#Tower et al., 2000 [17]	**Median BI (IQR) in mths:** CS failure to progress (32, 23–45); CS fetal distress (34, 25–46); SVD (32, 23–44)	No evidence that women delivering by Caesarean section have significantly longer waiting times to next pregnancy or birth
	**Median IPI (IQR) in mths:** CS failure to progress (28, 22–40); CS fetal distress (31, 24–44); SVD (29, 22–39)	
Hemminki, 1987 [37]	**BI:** Subsequent pregnancy within 5 years *1973 cohort-* RR (0.91, 95% CI 0.89, 0.93); *1976 cohort* RR (0.91, 95% CI 0.89, 1.12)	Proportion of women with a previous Caesarean section less likely to have a subsequent delivery, although no significant difference found
LaSala, 1987 [35]	**BI:** CS delivery (5.5%) >2 years without conceiving; vaginal delivery (1.4%) >2 years without conceiving	Women with a previous Caesarean were less likely to have a subsequent birth and took longer to conceive than women with a previous vaginal delivery. However the sample size was very small
Hemminki et al., 1985 [36]	**Median BI (mths):** CS delivery (44.4); vaginal delivery (45.6)	No significant difference in waiting time to next birth among women with a previous Caesarean section compared to women with a previous vaginal delivery

**Table Legend: *SVD*** Spontaneous vaginal delivery, ***CS*** Caesarean section, ***Mths*** Months, ***IPI*** Inter-pregnancy Interval,*** BI*** Birth Interval,*** RR*** Relative Risk,*** IQR*** Interquartile Range.

#Tower et al. reported both the median IPI and BI.

##### Birth interval (BI)

Three studies used BI as the measure of association between a Caesarean delivery and time to next birth [35-37]. One study reported a longer BI among women with a Caesarean delivery. La Sala et al. [35] reported that 5.5% of women with a Caesarean delivery compared to 1.4% of women with a vaginal delivery took greater than two years to conceive. Two studies negate such findings and reported no delay in time to next birth among women with a Caesarean delivery [36,37]. One study [17] reported both the IPI and BI and showed that no evidence existed to show that a Caesarean delivery was associated with a longer time to next pregnancy or birth.

### Heterogeneity assessment

The heterogeneity of the 11 included studies is presented in Table 1, where the different characteristics including sample size, region, population sampling and data sources are outlined. Ten of the 11 studies were cohort studies. Only one study [10] was conducted in a low-income developing country. In addition, the studies were conducted in varying time periods, including one study from the 1950’s to early 1980’s [36]. Seven studies collected information from single institutions or interviews and questionnaire data. The I^2^ statistic was used to quantify statistical heterogeneity and ranged from 0% to 88%, a high amount of heterogeneity between the studies. Definitions of pregnancy interval used varied between studies, with 44% heterogeneity in studies using BI compared to 45% heterogeneity in studies using IPI.

### Quality assessment

The studies varied in methodological quality (Table 4). According to the bias classification system used (Additional file 2), six were considered high in overall bias [17,32,33,35-37] due mainly to lack of adjustment for confounding or small sample size, [34] and five were considered low in bias [7,10,16,18,34]. The five studies considered low in bias and which adjusted for a minimum of three potential confounders including maternal age were included in the meta-analysis.

**Table 4:** Quality assessment of studies included in the systematic review

**Study**	**Selection bias**	**Exposure bias**	**Outcome assessment bias**	Confounding factor bias*	**Analytical bias**	**Attrition bias**	**Overall likelihood of bias**
Eijsink et al., 2008 [32]	Moderate	Low	Low	High (no adjustment for confounders reported, matched by maternal age and date of delivery)	Low	Minimal	High
Collin et al., 2006 [10]	Low	Low	Low	Minimal (adjusted for age, parity, level of education, urban or rural residence and young age at first intercourse)	Minimal	Low	Low
Smith et al., 2006 [18]	Minimal	Low	Low	Minimal (adjusted for marital status, deprivation, birth weight, infant sex, maternal age, maternal height and method of induction)	Minimal	Minimal	Low
Murphy et al., 2002 [7]	Low	Low	Low	Minimal (adjusted for maternal and paternal age, co-habitation, oral contraceptive pill use, cigarette exposure, alcohol consumption, educational level, ethnicity, parity, change of partner, maternal BMI)	Minimal	Minimal	Low
Zdeb et al., 1984 [33]	Low	Low	Low	Moderate (none reported). Matching by race, complications of pregnancy, maternal education and maternal age	Moderate	Low	High
Tollånes et al., 2007 [16]	Minimal	Low	Low	Minimal (stratified by maternal age, level of education and infant survival). Sub-group analyses by low-risk group, pre-eclampsia and breech presentation	Low	Minimal	Low
Tower et al., 2000 [16]	Low	Low	Low	Moderate (no adjustment for confounding). Matching by age and date of delivery	Moderate	Moderate	High
Huttly et al., 1990 [34]	Low	Low	Low	Minimal (adjusted for income, age, education and parity)	Moderate	Moderate	Low
Hemminki, 1987 [37]	Low	Low	Low	Moderate (no adjustment for confounders). Matching by year of birth, maternal age and infant sex	Moderate	Low	High
LaSala et al., 1987 [35]	Moderate	Low	Minimal	Moderate (no adjustment for confounding reported). Matching by age and parity	Moderate	Moderate	High
Hemminki et al., 1985 [36]	Low	Low	Minimal	Moderate (no adjustment reported). Matching by date of birth, mother’s age, race and marital status	Moderate	Minimal	High

Table Legend: *Assessment of confounding factor bias was done by evaluation of each study’s assessment of potential confounders by four methods: adjustment with regression, matching, assessment of potential confounders on univariate analyses that were found not to be significantly different between groups, and assessment of potential confounders on univariate analyses that were different between groups and not controlled for.

## Discussion

The overall findings of the meta-analysis suggest that there is a 10% increased risk of subsequent sub-fertility following Caesarean delivery compared to vaginal delivery. This finding persisted in the various sensitivity analyses undertaken including parity, publication date, length of follow-up, indication for mode of delivery, cohort size and by definition of sub-fertility used. However, it must be stated that the extent of subsequent sub-fertility was less pronounced among primiparous women (9%), studies published since 2000 (9%), and where the indication for mode of delivery was known (8%). The findings of this systematic review and meta-analysis are in agreement with a recent systematic review [12] which reported that patients who had undergone a Caesarean section had a 9% lower subsequent pregnancy rate [RR 0.91, 95% CI 0.87, 0.95] and an 11% lower birth rate [RR 0.89, 95% CI 0.87, 0.92], compared with women who delivered vaginally, and thus that previous Caesarean delivery was associated with an increased risk of subsequent sub-fertility. However, in contrast to the present systematic review, the authors focused on subsequent pregnancy rate following Caesarean delivery compared to vaginal delivery as the primary outcome measure of sub-fertility and did not include time to next pregnancy or birth in their meta-analysis. In addition, we identified two further papers for inclusion in the meta-analysis on pregnancy interval which were not included by Gurol-Urganci et al. [7,10].

Findings from the six studies included in the systematic review (but not the meta-analysis) produced conflicting results. It is possible that the findings reported in these studies are due to poor epidemiological methods used, including small sample size, inability to adjust for confounding, no indication for mode of delivery, and significant variations in the measurement of the outcome variable (time to next pregnancy or birth). However, from the meta-analysis it can be seen that the studies which found an association between Caesarean delivery and a longer waiting time to next pregnancy or birth [10,16,18] were of a more methodologically superior quality, including large sample size, population-based registries and had detailed obstetrical information including the indication for Caesarean delivery, maternal obstetric history and a long period of follow-up.

It can be argued however that residual or unknown confounders or maternal choice to intentionally delay next birth may explain any delay in waiting time to next birth and that the sub-fertility observed may not be as a result of Caesarean delivery [16,18]. According to Bhattacharya et al. [38] voluntary factors responsible for intentionally delayed subsequent pregnancies include social and financial factors such as lifestyle, age, educational attainment and lack of a partner. Previous experience of labour and delivery are also vital factors affecting the decision to delay or avoid another pregnancy in women. This view is also discussed by Porter et al. [13] and Kjerulff et al. [39] both of whom argue that the mechanism behind subsequent sub-fertility could be social or psychological rather than pathological.

### Strengths and limitations of the review

Strengths of the review include the thoroughness of the literature search which included seven databases, two reviewers, a detailed search-strategy compiled with a team of obstetricians and epidemiologists and rigorous cross-checking of reference lists. Furthermore, no date or language restrictions were enforced. Unfortunately, limitations to this review include firstly that any systematic review is limited by the quality of the original data collected, especially with observational studies. For example, only five out of the 11 included studies adjusted for potential confounders [7,10,16,18,34], and the other six studies used matching to reduce the likelihood of bias [17,32,33,35-37]. It was not possible to meta-analyse the six studies which only presented crude estimates as each reported pregnancy interval using very different measures making it impossible to combine the estimates. Sample sizes were quite small in three of the studies that reported an association [32,35,36] and thus the findings may be due to chance. Furthermore, the indication for mode of delivery (instrumental vaginal, vaginal breech, elective caesarean section, emergency caesarean section, etc.) was available in only two of the meta-analysed studies [16,18] and so confounding by indication may also exist in some of the included studies. Short periods of follow-up may also be an issue, as some women were followed for short periods including: one year [36], three years [35], four years [34] and five years [17,33,37], which may not be a sufficient amount of time to allow for a subsequent pregnancy in some cases. The loss of a child and the desire to replace the loss is another important factor that is not acknowledged in all studies, with only one study stratifying analyses based on infant survival or death within the first year [16].

Finally, to know whether there is any association between Caesarean delivery and subsequent sub-fertility, it would be necessary to know whether women were deliberately delaying pregnancy (difficult deliveries in the past, desired family size obtained, oral contraceptive use, varying recommendations by doctors on the optimal time to wait following a Caesarean delivery before trying for another baby again, or change of partner). It is also not possible to establish whether any fertility treatment was used by the study populations. This in itself would be a major confounder as a woman who was already sub-fertile may have an increased risk of sub-fertility in the future. Ideally, subsequent research using registry data for instance, should aim to access the fertility registers (available in the Scandinavian countries for example) in order to exclude or account for certain groups such as women with a history of poor reproductive outcomes or women who accessed fertility treatments such as *in vitro* fertilisation (IVF). Sub-group analyses by other important risk factors such as advanced maternal age should also be prioritised. The main focus should be on the likelihood and timing of a subsequent pregnancy in women with a previous Caesarean section compared to vaginal delivery where no adverse outcomes occur (i.e. in live births only) in order to minimise the bias of pre-existing sub-fertility. In this review, only three studies used a cohort of women actively trying to conceive again [7,10,36]. It was possible to include two out these three studies in a sub-group analysis of women actively trying to conceive, generating a pooled OR of 0.82 (95% CI 0.72, 0.94) as the rate of subsequent sub-fertility. Secondly, only published literature was included in this systematic review and so publication bias may be cited as a reason for insufficient validity. It is however unlikely that any large study was missed due to the thoroughness of the literature search.

### Methodological issues in measuring time to pregnancy

Much of the research to date investigating a woman’s fertility has been based on time to pregnancy studies, with retrospective designs using a well-defined target population, which are cheaper to conduct with the added benefit of higher external validity compared to prospective studies [40]. However, methodological issues exist in time to pregnancy studies including the inability to obtain detailed time-specific information about behaviour and risk factors [41]. Appropriate study design can minimise the biases associated with time to pregnancy studies according to Joffe et al. [40], who describe three main sampling frames: 1) *pregnancy-based studies* recruiting pregnant or recently delivered women who are easy to define, gain access to and recall bias is minimal, but sterile couples are excluded and sub-fertile couples under-represented; 2) *cross-sectional population-based or occupationally-based studies* where couples are randomly selected from the general population or an occupational group and a detailed obstetric history can be retrieved on one or more pregnancies, and failed attempts. However, long recall may lead to possible bias, for exposure variables and covariates; 3) *population-based birth cohort* studies use a previously defined birth cohort, who are questioned about their reproductive history, allowing for a longitudinal aspect of factors from earlier life to be studied. The sampling frames of each study included in the systematic review can be found in the supplementary material (Additional file 3).

Different biases including selection bias (including successful pregnancy attempts only), ‘wantedness’ bias (where couples say that a pregnancy was planned when it was not for fear of appearing not to want the child), planning bias (excluding couples with accidental or unplanned pregnancies), and pregnancy recognition bias (where a pregnancy is identified very early into the gestation may result in miscarriage in some cases) can be controlled if studies are well-designed and conducted, and through the use of appropriate sensitivity analyses in properly selected populations [42]. Time to pregnancy data has shown to be valid even where there is a recall of 20 years, although non-birth outcomes such as miscarriage are more commonly excluded [40,43-45].

## Conclusions

Based on the findings of the studies included in this meta-analysis, previous Caesarean delivery is associated with an increase in subsequent sub-fertility (i.e. a delay in time to next pregnancy or birth) compared to vaginal delivery by as much as 14%. With Caesarean rates at the highest ever recorded, the average age of first-time mothers increasing gradually and primary elective Caesarean delivery common practice, it is important that clinicians and expectant mothers fully discuss the potential benefits and risks associated with not only Caesarean deliveries, but all modes of delivery and together make an informed decision on an individual basis. [13,46]. The underlying mechanisms for an association between Caesarean delivery and subsequent sub-fertility remains unclear and for this reason, there is an urgent need for prospective research with large sample sizes and appropriate acknowledgement of potential key confounders. Further research is needed with a more thorough approach using a combination of quantitative and qualitative methods to address women’s views of their experiences [47-49]. Even if an increased waiting time to next pregnancy or birth is reported it could still be due to biological or psychological factors and therefore, the woman’s opinion about the length of acceptable pregnancy interval is perhaps the most important predicting factor and should be incorporated into future research where possible.

## Competing interests

The authors declare that they have no competing interests.

## Authors’ contributions

SMON, PMK, LCK, ASK, RAG and TBH conceived and designed the study. SMON and ASK acquired, analysed and interpreted the data. SMON drafted the manuscript. PMK, LCK, ASK, JEL, RAG and TBH critically revised the manuscript for important intellectual content. SMON will act as guarantor for the paper. All authors read and approved the final manuscript.

## Pre-publication history

The pre-publication history for this paper can be accessed here:

http://www.biomedcentral.com/1471-2393/13/165/prepub

## Supplementary Material

Additional file 1 **Comprehensive list of search terms used.** List of all search terms used for each database. Click here for file

Additional file 2 **Bias classification tool – quality assessment.** Tool used to assess amount of bias present in the included studies for quality assessment. Click here for file

Additional file 3 **Sampling frame used by the studies included in the systematic review.** Table detailing the sampling frame used by each study. Click here for file
