# Supplementary material for: Caesarean delivery and subsequent pregnancy interval: a systematic review and meta-analysis
Source: BMC Pregnancy Childbirth. 2013 Aug 27;13:165. doi: 10.1186/1471-2393-13-165 (PMC3765853; doi:10.1186/1471-2393-13-165)
Supplement: Additional file 1 — Comprehensive list of search terms used. List of all search terms used for each database. [file 1471-2393-13-165-S1.doc]

| **Search terms** | **No of records returned** |
| --- | --- |
| ***For CINAHL (1981- October 17th 2012 ) LIMIT: humans*** |  |
| 1. Caesarean section | 3351 |
| 1. Caesarean sections | 728 |
| 1. Cesarean section | 8690 |
| 1. Cesarean sections | 841 |
| 1. Delivery, abdominal | 106 |
| 1. Deliveries, abdominal | 17 |
| 1. Abdominal delivery | 106 |
| 1. Abdominal deliveries | 17 |
| 1. C-section | 1023 |
| 1. C-sections | 325 |
| 1. C section | 3365 |
| 1. C sections | 1008 |
| 1. Postcesarean section | 12 |
| 1. Postcaesarean section | 3 |
| 1. Caesarean delivery | 975 |
| 1. Cesarean delivery | 3518 |
| 1. Delivery, Caesarean | 975 |
| 1. Delivery, Cesarean | 3518 |
| 1. Mode of delivery | 2213 |
| 1. [#1 or #2 or #3 or #4 or #5 or #6 or #7 or #8 or #9 or #10 or #11 or #12 or #13 or #14 or #15 or #16 or #17 or #18 or #19] | 17332 |
| 1. Birth interval | 168 |
| 1. Birth intervals | 185 |
| 1. Birth spacing | 36 |
| 1. Birth spacings | 0 |
| 1. Pregnancy interval | 95 |
| 1. Pregnancy intervals | 34 |
| 1. First birth interval | 12 |
| 1. First birth intervals | 4 |
| 1. Inter delivery interval | 1484 |
| 1. Inter delivery intervals | 3288 |
| 1. Inter-delivery interval | 1484 |
| 1. Inter-delivery intervals | 3288 |
| 1. Between pregnancy interval | 13 |
| 1. Between pregnancy intervals | 1 |
| 1. Inter pregnancy interval | 3 |
| 1. Inter pregnancy intervals | 5 |
| 1. Inter-pregnancy interval | 3 |
| 1. Inter-pregnancy intervals | 5 |
| 1. Subsequent pregnancy interval | 1 |
| 1. Subsequent pregnancy intervals | 3686 |
| 1. Pregnancy spacing | 12 |
| 1. Pregnancy spacings | 0 |
| 1. Pregnancy gap | 18 |
| 1. Pregnancy gaps | 9 |
| 1. First birth spacing | 1 |
| 1. First birth spacings | 2 |
| 1. Timing between pregnancy | 3 |
| 1. Inter gestational time | 1836 |
| 1. [#21 or #22 or #23 or #24 or #25 or #26 or #27 or #28 or #29 or #30 or #31 or #32 or #33 or #34 or #35 or #36 or #37 or #38 or #39 or #40 or #41 or #42 or #43 or #44 or #45 or #46 or #47 or #48] | 9591 |
| 1. [#20 and #49] | 1229 |
| 1. Article | 118753 |
| 1. Cohort study | 17449 |
| 1. Case control study | 6109 |
| 1. Systematic review | 18084 |
| 1. Review | 155756 |
| 1. Retrospective study | 13539 |
| 1. Prospective study | 26128 |
| 1. Cross sectional study |  |
| 1. [#51 or #52 or #53 or #54 or #55 or #56 or #57] | 302320 |
| 1. Female |  |
| 1. Woman |  |
| 1. Human |  |
| 1. Longterm effects | 30 |
| 1. Long-term effects | 3796 |
| 1. Long-term complications | 1118 |
| 1. Longterm complications | 13 |
| 1. Pregnancy outcome | 1337 |
| 1. Postoperative complications | 18764 |
| 1. Subfertility | 230 |
| 1. Sub-fertility | 11 |
| 1. Subsequent fertility | 34 |
| 1. Secondary subfertility | 2 |
| 1. [#59 or #60 or #61 or #62 or #63 or #64 or #65 or #66 or #67 or #68] | 25121 |
| 1. **[#20 and #49 and #58 and #69]** | **2049** |

| **Search terms** | **No of records returned** |
| --- | --- |
| ***For PubMed (1966- October 17^th^ 2012 ) LIMIT: humans*** |  |
| 1. Caesarean section | 36773 |
| 1. Caesarean sections | 34515 |
| 1. Cesarean section | 36773 |
| 1. Cesarean sections | 34514 |
| 1. Delivery, abdominal | 36016 |
| 1. Deliveries, abdominal | 33860 |
| 1. Abdominal delivery | 36016 |
| 1. Abdominal deliveries | 33860 |
| 1. C-section | 33724 |
| 1. C-sections | 33618 |
| 1. C section | 33724 |
| 1. C sections | 33618 |
| 1. Postcesarean section | 33600 |
| 1. Postcaesarean section | 15 |
| 1. Caesarean delivery | 35754 |
| 1. Cesarean delivery | 35737 |
| 1. Delivery, Caesarean | 8845 |
| 1. Delivery, Cesarean | 33403 |
| 1. Mode of delivery | 5451 |
| 1. [#1 or #2 or #3 or #4 or #5 or #6 or #7 or #8 or #9 or #10 or #11 or #12 or #13 or #14 or #15 or #16 or #17 or #18 or #19] | 45797 |
| 1. Birth interval | 13899 |
| 1. Birth intervals | 5313 |
| 1. Birth spacing | 5584 |
| 1. Birth spacings | 5315 |
| 1. Pregnancy interval | 19875 |
| 1. Pregnancy intervals | 9882 |
| 1. First birth interval | 7720 |
| 1. First birth intervals | 5313 |
| 1. Inter delivery interval | 55 |
| 1. Inter delivery intervals | 30 |
| 1. Inter-delivery interval | 1 |
| 1. Inter-delivery intervals | 55 |
| 1. Between pregnancy interval | 19875 |
| 1. Between pregnancy intervals | 9882 |
| 1. Inter pregnancy interval | 145 |
| 1. Inter pregnancy intervals | 110 |
| 1. Inter-pregnancy interval | 27 |
| 1. Inter-pregnancy intervals | 19 |
| 1. Subsequent pregnancy interval | 1299 |
| 1. Subsequent pregnancy intervals | 594 |
| 1. Pregnancy spacing | 428 |
| 1. Pregnancy spacings | 3 |
| 1. Pregnancy gap | 889 |
| 1. Pregnancy gaps | 586 |
| 1. First birth spacing | 209 |
| 1. First birth spacings | 2 |
| 1. Timing between pregnancy | 3778 |
| 1. Inter gestational time | 79 |
| 1. [#21 or #22 or #23 or #24 or #25 or #26 or #27 or #28 or #29 or #30 or #31 or #32 or #33 or #34 or #35 or #36 or #37 or #38 or #39 or #40 or #41 or #42 or #43 or #44 or #45 or #46 or #47 or #48] | 32179 |
| 1. [#20 and #49] | 3458 |
| 1. Article | 10965986 |
| 1. Cohort study | 1162626 |
| 1. Case control study | 567715 |
| 1. Systematic review | 1438238 |
| 1. Review | 1669141 |
| 1. Retrospective study | 428389 |
| 1. Prospective study | 388037 |
| 1. Cross sectional study |  |
| 1. [#51 or #52 or #53 or #54 or #55 or #56 or #57] | 11026029 |
| 1. Female |  |
| 1. Woman |  |
| 1. Human |  |
| 1. Longterm effects | 1519582 |
| 1. Long-term effects | 1604867 |
| 1. Long-term complications | 90443 |
| 1. Longterm complications | 1324 |
| 1. Pregnancy outcome | 68444 |
| 1. Postoperative complications | 392802 |
| 1. Subfertility | 50969 |
| 1. Sub-fertility | 50732 |
| 1. Subsequent fertility | 1791 |
| 1. Secondary subfertility | 2051 |
| 1. [#59 or #60 or #61 or #62 or #63 or #64 or #65 or #66 or #67 or #68] | 2032274 |
| 1. **[#20 and #49 and #58 and #69]** | **2378** |

| **Search terms** | **No of records returned** |
| --- | --- |
| ***For Medline (1966- October 17^th^ 2012 ) LIMIT: humans*** |  |
| 1. Caesarean section | 11360 |
| 1. Caesarean sections | 1544 |
| 1. Cesarean section | 38613 |
| 1. Cesarean sections | 3337 |
| 1. Delivery, abdominal | 3825 |
| 1. Deliveries, abdominal | 500 |
| 1. Abdominal delivery | 3825 |
| 1. Abdominal deliveries | 500 |
| 1. C-section | 469 |
| 1. C-sections | 148 |
| 1. C section | 28491 |
| 1. C sections | 14287 |
| 1. Postcesarean section | 246 |
| 1. Postcaesarean section | 16 |
| 1. Caesarean delivery | 5497 |
| 1. Cesarean delivery | 17044 |
| 1. Delivery, Caesarean | 5497 |
| 1. Delivery, Cesarean | 17044 |
| 1. Mode of delivery | 6649 |
| 1. [#1 or #2 or #3 or #4 or #5 or #6 or #7 or #8 or #9 or #10 or #11 or #12 or #13 or #14 or #15 or #16 or #17 or #18 or #19] | 93997 |
| 1. Birth interval | 11475 |
| 1. Birth intervals | 6960 |
| 1. Birth spacing | 1047 |
| 1. Birth spacings | 4 |
| 1. Pregnancy interval | 18332 |
| 1. Pregnancy intervals | 10344 |
| 1. First birth interval | 3366 |
| 1. First birth intervals | 2156 |
| 1. Inter delivery interval | 78 |
| 1. Inter delivery intervals | 52 |
| 1. Inter-delivery interval | 1 |
| 1. Inter-delivery intervals | 1 |
| 1. Between pregnancy interval | 9934 |
| 1. Between pregnancy intervals | 4933 |
| 1. Inter pregnancy interval | 145 |
| 1. Inter pregnancy intervals | 124 |
| 1. Inter-pregnancy interval | 30 |
| 1. Inter-pregnancy intervals | 21 |
| 1. Subsequent pregnancy interval | 1319 |
| 1. Subsequent pregnancy intervals | 703 |
| 1. Pregnancy spacing | 652 |
| 1. Pregnancy spacings | 3 |
| 1. Pregnancy gap | 1387 |
| 1. Pregnancy gaps | 685 |
| 1. First birth spacing | 230 |
| 1. First birth spacings | 2 |
| 1. Timing between pregnancy | 1894 |
| 1. Inter gestational time | 87 |
| 1. [#21 or #22 or #23 or #24 or #25 or #26 or #27 or #28 or #29 or #30 or #31 or #32 or #33 or #34 or #35 or #36 or #37 or #38 or #39 or #40 or #41 or #42 or #43 or #44 or #45 or #46 or #47 or #48] | 38445 |
| 1. [#20 and #49] | 3369 |
| 1. Article | 329145 |
| 1. Cohort study | 161878 |
| 1. Case control study | 141390 |
| 1. Systematic review | 45784 |
| 1. Review | 948279 |
| 1. Retrospective study | 249597 |
| 1. Prospective study | 292088 |
| 1. [#51 or #52 or #53 or #54 or #55 or #56 or #57] | 1865932 |
| 1. Longterm effects | 2385 |
| 1. Long-term effects | 177486 |
| 1. Long-term complications | 92492 |
| 1. Longterm complications | 1378 |
| 1. Pregnancy outcome | 71444 |
| 1. Postoperative complications | 332025 |
| 1. Subfertility | 1896 |
| 1. Sub-fertility | 99 |
| 1. Subsequent fertility | 4074 |
| 1. Secondary subfertility | 115 |
| 1. [#59 or #60 or #61 or #62 or #63 or #64 or #65 or #66 or #67 or #68] | 617920 |
| 1. **[#20 and #49 and #58 and #69]** | **2366** |

| **Search terms** | **No of records returned** |
| --- | --- |
| ***For Embase (1974- October 17^th^ 2012 ) LIMIT: humans*** |  |
| 1. Caesarean section | 15706 |
| 1. Caesarean sections | 2182 |
| 1. Cesarean section | 56415 |
| 1. Cesarean sections | 4720 |
| 1. Delivery, abdominal | 129898 |
| 1. Deliveries, abdominal | 1282 |
| 1. Abdominal delivery | 129898 |
| 1. Abdominal deliveries | 1282 |
| 1. C-section | 786 |
| 1. C-sections | 221 |
| 1. C section | 169945 |
| 1. C sections | 57320 |
| 1. Postcesarean section | 303 |
| 1. Postcaesarean section | 27 |
| 1. Caesarean delivery | 14945 |
| 1. Cesarean delivery | 55647 |
| 1. Delivery, Caesarean | 14945 |
| 1. Delivery, Cesarean | 55647 |
| 1. Mode of delivery | 19064 |
| 1. [#1 or #2 or #3 or #4 or #5 or #6 or #7 or #8 or #9 or #10 or #11 or #12 or #13 or #14 or #15 or #16 or #17 or #18 or #19] | 399408 |
| 1. Birth interval | 18287 |
| 1. Birth intervals | 7518 |
| 1. Birth spacing | 945 |
| 1. Birth spacings | 5 |
| 1. Pregnancy interval | 24399 |
| 1. Pregnancy intervals | 9918 |
| 1. First birth interval | 5111 |
| 1. First birth intervals | 2308 |
| 1. Inter delivery interval | 514 |
| 1. Inter delivery intervals | 276 |
| 1. Inter-delivery interval | 4 |
| 1. Inter-delivery intervals | 1 |
| 1. Between pregnancy interval | 13074 |
| 1. Between pregnancy intervals | 4766 |
| 1. Inter pregnancy interval | 224 |
| 1. Inter pregnancy intervals | 145 |
| 1. Inter-pregnancy interval | 42 |
| 1. Inter-pregnancy intervals | 19 |
| 1. Subsequent pregnancy interval | 1617 |
| 1. Subsequent pregnancy intervals | 608 |
| 1. Pregnancy spacing | 648 |
| 1. Pregnancy spacings | 2 |
| 1. Pregnancy gap | 1685 |
| 1. Pregnancy gaps | 913 |
| 1. First birth spacing | 185 |
| 1. First birth spacings | 2 |
| 1. Timing between pregnancy | 2450 |
| 1. Inter gestational time | 118 |
| 1. [#21 or #22 or #23 or #24 or #25 or #26 or #27 or #28 or #29 or #30 or #31 or #32 or #33 or #34 or #35 or #36 or #37 or #38 or #39 or #40 or #41 or #42 or #43 or #44 or #45 or #46 or #47 or #48] | 47605 |
| 1. [#20 and #49] | 5057 |
| 1. Article | 24423441 |
| 1. Cohort study | 257876 |
| 1. Case control study | 361495 |
| 1. Systematic review | 97255 |
| 1. Review | 2531587 |
| 1. Retrospective study | 398595 |
| 1. Prospective study | 427866 |
| 1. [#51 or #52 or #53 or #54 or #55 or #56 or #57] | 24463385 |
| 1. Longterm effects | 2485 |
| 1. Long-term effects | 177237 |
| 1. Long-term complications | 110216 |
| 1. Longterm complications | 1283 |
| 1. Pregnancy outcome | 92962 |
| 1. Postoperative complications | 158278 |
| 1. Subfertility | 3584 |
| 1. Sub-fertility | 177 |
| 1. Subsequent fertility | 7643 |
| 1. Secondary subfertility | 197 |
| 1. [#59 or #60 or #61 or #62 or #63 or #64 or #65 or #66 or #67 or #68] | 489387 |
| 1. **[#20 and #49 and #58 and #69]** | **2181** |

| **Search terms** | **No of records returned** |
| --- | --- |
| ***For Web of Knowledge (1945- October 17^th^ 2012 ) LIMIT: humans*** |  |
| 1. Caesarean section | 81129 |
| 1. Caesarean sections | 81162 |
| 1. Cesarean section | 81654 |
| 1. Cesarean sections | 81654 |
| 1. Delivery, abdominal | 9112 |
| 1. Deliveries, abdominal | 9112 |
| 1. Abdominal delivery | 9112 |
| 1. Abdominal deliveries | 9112 |
| 1. C-section | 964 |
| 1. C-sections | 964 |
| 1. C section | 170655 |
| 1. C sections | 170655 |
| 1. Postcesarean section | 397 |
| 1. Postcaesarean section | 21 |
| 1. Caesarean delivery | 46392 |
| 1. Cesarean delivery | 46591 |
| 1. Delivery, Caesarean | 46367 |
| 1. Delivery, Cesarean | 46591 |
| 1. Mode of delivery | 19883 |
| 1. [#1 or #2 or #3 or #4 or #5 or #6 or #7 or #8 or #9 or #10 or #11 or #12 or #13 or #14 or #15 or #16 or #17 or #18 or #19] | 278985 |
| 1. Birth interval | 42184 |
| 1. Birth intervals | 42184 |
| 1. Birth spacing | 7204 |
| 1. Birth spacings | 1436 |
| 1. Pregnancy interval | 50852 |
| 1. Pregnancy intervals | 50823 |
| 1. First birth interval | 12684 |
| 1. First birth intervals | 12693 |
| 1. Inter delivery interval | 189 |
| 1. Inter delivery intervals | 189 |
| 1. Inter-delivery interval | 4 |
| 1. Inter-delivery intervals | 4 |
| 1. Between pregnancy interval | 26594 |
| 1. Between pregnancy intervals | 26576 |
| 1. Inter pregnancy interval | 330 |
| 1. Inter pregnancy intervals | 330 |
| 1. Inter-pregnancy interval | 73 |
| 1. Inter-pregnancy intervals | 73 |
| 1. Subsequent pregnancy interval | 3789 |
| 1. Subsequent pregnancy intervals | 3789 |
| 1. Pregnancy spacing | 7714 |
| 1. Pregnancy spacings | 817 |
| 1. Pregnancy gap | 3472 |
| 1. Pregnancy gaps | 3470 |
| 1. First birth spacing | 1114 |
| 1. First birth spacings | 294 |
| 1. Timing between pregnancy | 54280 |
| 1. Inter gestational time | 137 |
| 1. [#21 or #22 or #23 or #24 or #25 or #26 or #27 or #28 or #29 or #30 or #31 or #32 or #33 or #34 or #35 or #36 or #37 or #38 or #39 or #40 or #41 or #42 or #43 or #44 or #45 or #46 or #47 or #48] | 134583 |
| 1. [#20 and #49] | 10513 |
| 1. Article | 1112937 |
| 1. Cohort study | 502390 |
| 1. Case control study | 656134 |
| 1. Systematic review | 131986 |
| 1. Review | 2686271 |
| 1. Retrospective study | 661545 |
| 1. Prospective study | 776823 |
| 1. [#51 or #52 or #53 or #54 or #55 or #56 or #57] | 5426753 |
| 1. Longterm effects | 7547 |
| 1. Long-term effects | 524065 |
| 1. Long-term complications | 162792 |
| 1. Longterm complications | 2482 |
| 1. Pregnancy outcome | 144619 |
| 1. Postoperative complications | 432355 |
| 1. Subfertility | 5123 |
| 1. Sub-fertility | 185 |
| 1. Subsequent fertility | 10597 |
| 1. Secondary subfertility | 172 |
| 1. [#59 or #60 or #61 or #62 or #63 or #64 or #65 or #66 or #67 or #68] | 1180789 |
| 1. **[#20 and #49 and #58 and #69]** | **2955** |

| **Search terms** | **No of records returned** |
| --- | --- |
| ***For Scopus (1960- October 17^th^ 2012 ) LIMIT: humans*** |  |
| 1. Caesarean section | 26209 |
| 1. Caesarean sections | 14301 |
| 1. Cesarean section | 56642 |
| 1. Cesarean sections | 56642 |
| 1. Delivery, abdominal | 6977 |
| 1. Deliveries, abdominal | 6977 |
| 1. Abdominal delivery | 6977 |
| 1. Abdominal deliveries | 6977 |
| 1. C-section | 1017 |
| 1. C-sections | 1017 |
| 1. C section | 205902 |
| 1. C sections | 205902 |
| 1. Postcesarean section | 311 |
| 1. Postcaesarean section | 25 |
| 1. Caesarean delivery | 7530 |
| 1. Cesarean delivery | 26903 |
| 1. Delivery, Caesarean | 7530 |
| 1. Delivery, Cesarean | 26903 |
| 1. Mode of delivery | 13043 |
| 1. [#1 or #2 or #3 or #4 or #5 or #6 or #7 or #8 or #9 or #10 or #11 or #12 or #13 or #14 or #15 or #16 or #17 or #18 or #19] | 19095205 |
| 1. Birth interval | 20985 |
| 1. Birth intervals | 20985 |
| 1. Birth spacing | 1334 |
| 1. Birth spacings | 1334 |
| 1. Pregnancy interval | 30730 |
| 1. Pregnancy intervals | 30730 |
| 1. First birth interval | 6317 |
| 1. First birth intervals | 6317 |
| 1. Inter delivery interval | 173 |
| 1. Inter delivery intervals | 173 |
| 1. Inter-delivery interval | 4 |
| 1. Inter-delivery intervals | 4 |
| 1. Between pregnancy interval | 15421 |
| 1. Between pregnancy intervals | 15421 |
| 1. Inter pregnancy interval | 282 |
| 1. Inter pregnancy intervals | 282 |
| 1. Inter-pregnancy interval | 44 |
| 1. Inter-pregnancy intervals | 44 |
| 1. Subsequent pregnancy interval | 2072 |
| 1. Subsequent pregnancy intervals | 2072 |
| 1. Pregnancy spacing | 820 |
| 1. Pregnancy spacings | 820 |
| 1. Pregnancy gap | 2495 |
| 1. Pregnancy gaps | 2495 |
| 1. First birth spacing | 292 |
| 1. First birth spacings | 292 |
| 1. Timing between pregnancy | 6219 |
| 1. Inter gestational time | 138 |
| 1. [#21 or #22 or #23 or #24 or #25 or #26 or #27 or #28 or #29 or #30 or #31 or #32 or #33 or #34 or #35 or #36 or #37 or #38 or #39 or #40 or #41 or #42 or #43 or #44 or #45 or #46 or #47 or #48] | 8577583 |
| 1. [#20 and #49] | 32559 |
| 1. Article | 16809245 |
| 1. Cohort study | 267456 |
| 1. Case control study | 391139 |
| 1. Systematic review | 92060 |
| 1. Review | 2829705 |
| 1. Retrospective study | 504987 |
| 1. Prospective study | 469519 |
| 1. [#51 or #52 or #53 or #54 or #55 or #56 or #57] | 2367013 |
| 1. Longterm effects | 4381 |
| 1. Long-term effects | 259403 |
| 1. Long-term complications | 76694 |
| 1. Longterm complications | 1712 |
| 1. Pregnancy outcome | 101416 |
| 1. Postoperative complications | 416857 |
| 1. Subfertility | 3300 |
| 1. Sub-fertility | 149 |
| 1. Subsequent fertility | 4500 |
| 1. Secondary subfertility | 167 |
| 1. [#59 or #60 or #61 or #62 or #63 or #64 or #65 or #66 or #67 or #68] | 2538971 |
| 1. **[#20 and #49 and #58 and #69]** | **554** |

| **Search terms** | **No of records returned** |
| --- | --- |
| ***For The Cochrane Library (1993- October 17^th^ 2012 ) LIMIT: humans*** |  |
| 1. Caesarean section | 2171 |
| 1. Caesarean sections | 1582 |
| 1. Cesarean section | 3413 |
| 1. Cesarean sections | 3413 |
| 1. Delivery, abdominal | 251 |
| 1. Deliveries, abdominal | 251 |
| 1. Abdominal delivery | 251 |
| 1. Abdominal deliveries | 251 |
| 1. C-section | 33 |
| 1. C-sections | 33 |
| 1. C section | 469 |
| 1. C sections | 469 |
| 1. Postcesarean section | 29 |
| 1. Postcaesarean section | 3 |
| 1. Caesarean delivery | 602 |
| 1. Cesarean delivery | 1450 |
| 1. Delivery, Caesarean | 602 |
| 1. Delivery, Cesarean | 1450 |
| 1. Mode of delivery | 696 |
| 1. [#1 or #2 or #3 or #4 or #5 or #6 or #7 or #8 or #9 or #10 or #11 or #12 or #13 or #14 or #15 or #16 or #17 or #18 or #19] | 5936 |
| 1. Birth interval | 3996 |
| 1. Birth intervals | 3996 |
| 1. Birth spacing | 414 |
| 1. Birth spacings | 0 |
| 1. Pregnancy interval | 4740 |
| 1. Pregnancy intervals | 4740 |
| 1. First birth interval | 3452 |
| 1. First birth intervals | 3452 |
| 1. Inter delivery interval | 304 |
| 1. Inter delivery intervals | 304 |
| 1. Inter-delivery interval | 0 |
| 1. Inter-delivery intervals | 0 |
| 1. Between pregnancy interval | 3544 |
| 1. Between pregnancy intervals | 3544 |
| 1. Inter pregnancy interval | 250 |
| 1. Inter pregnancy intervals | 250 |
| 1. Inter-pregnancy interval | 4 |
| 1. Inter-pregnancy intervals | 4 |
| 1. Subsequent pregnancy interval | 1068 |
| 1. Subsequent pregnancy intervals | 1068 |
| 1. Pregnancy spacing | 444 |
| 1. Pregnancy spacings | 0 |
| 1. Pregnancy gap | 221 |
| 1. Pregnancy gaps | 221 |
| 1. First birth spacing | 392 |
| 1. First birth spacings | 0 |
| 1. Timing between pregnancy | 4752 |
| 1. Inter gestational time | 64 |
| 1. [#21 or #22 or #23 or #24 or #25 or #26 or #27 or #28 or #29 or #30 or #31 or #32 or #33 or #34 or #35 or #36 or #37 or #38 or #39 or #40 or #41 or #42 or #43 or #44 or #45 or #46 or #47 or #48] | 9367 |
| 1. [#20 and #49] | 1644 |
| 1. Article | 731959 |
| 1. Cohort study | 18545 |
| 1. Case control study | 54726 |
| 1. Systematic review | 30101 |
| 1. Review | 113702 |
| 1. Retrospective study | 12135 |
| 1. Prospective study | 96203 |
| 1. [#51 or #52 or #53 or #54 or #55 or #56 or #57] | 731959 |
| 1. Longterm effects | 6597 |
| 1. Long-term effects | 29911 |
| 1. Long-term complications | 1558 |
| 1. Longterm complications | 9716 |
| 1. Pregnancy outcome | 10044 |
| 1. Postoperative complications | 21463 |
| 1. Subfertility | 361 |
| 1. Sub-fertility | 28 |
| 1. Subsequent fertility | 363 |
| 1. Secondary subfertility | 228 |
| 1. [#59 or #60 or #61 or #62 or #63 or #64 or #65 or #66 or #67 or #68] | 59971 |
| 1. **[#20 and #49 and #58 and #69]** | **1175** |
