# Supplementary material for: Caesarean delivery and subsequent pregnancy interval: a systematic review and meta-analysis
Source: BMC Pregnancy Childbirth. 2013 Aug 27;13:165. doi: 10.1186/1471-2393-13-165 (PMC3765853; doi:10.1186/1471-2393-13-165)
Supplement: Additional file 2 — Bias classification tool – quality assessment. Tool used to assess amount of bias present in the included studies for quality assessment. [file 1471-2393-13-165-S2.doc]

| **Bias** | **NR** | **Minimal** | **Low** | **Moderate** | **High** |
| --- | --- | --- | --- | --- | --- |
| **Selection** |  | - Consectutive unselected population - Sample selected from general population rather than a select group - Eligibility criteria explained - Rational for case and control selection explained - Follow-up or assessment time explained | - Sample selected from large population but selection criteria not defined - A select group of population (based on race, ethnicity, residence, etc.) studied | - Sample selection ambiguous but sample may be representative - Eligibility criteria not explained - Rationale for cases and controls not explained - Follow-up or assessment time not explained | - Sample selection ambiguous and sample likely not representative - Comparative groups differ in baseline characteristics - A very select population studied making it difficult to generalise findings |
| **Exposure** |  | - Direct questioning (interview) or completion of survey by mother at the time of exposure or close to the time of exposure - Direct measurment of exposure (laboratory) - Exposure from the chart | - Assessment of exposure from a dataset - Indirect assessment (postal survey, mailed questionnaire) - Recall <1 year after birth | - Recall 1-5 years after birth - Extrapolating data from population exposure sample (with some assumptions) and not direct assessment at any time | - Recall >5 years after birth - Indirect method of assessment (obtaining data from others and not from mother or father) |
| **Outcome** |  | - Assessment from hospital record, birth certificate or from direct questionion of mothers about outcomes | - Assessment from administrative database | - Assessment from “close-ended” questions (Did you have a subsequent pregnancy?) | - Assessment from non-validated sources or generic estimate from overall population |
| **Confounding** |  | - Assessed for common confounders | - Only certain confounders assessed | - Not assessed for confounders |  |
| **Analytical** |  | - Analyses appropriate for type of sample (if matched: paired t test, McNemar) - Analytical method accounted for sampling strategy in cross-sectional study - Sample size calculation performed and adequate sample studied | - Analyses not accounting for common statistical adjustment (e.g. multiple analyses e.g. Bonferroni) when appropriate - Sample size calculation not performed, but all available eligible patients studied - Sample size calculated and reasons for not meeting sample size given | - Sample size estimation unclear or only sub-sample of eligible patients studied | - Analyses inappropriate for type of sample/study |
| **Attrition** |  | - None or <10% attrition and reasons for loss of follow-up explained - All subjects from initiation of study to final outcome assessment were accounted for | - <10% attrition AND reasons for loss of follow-up not explained - 11-20% attrition, reasons for loss of follow-up explained | - 11-20% attrition but reasons for loss of follow-up not explained - >20% attrition but reasons for loss of follow-up explained - All subjects from initiation of study to final outcome assessment not accounted for | - >20% attrition, reasons for loss of follow-up not explained |
