# Supplementary material for: Caesarean delivery and subsequent pregnancy interval: a systematic review and meta-analysis
Source: BMC Pregnancy Childbirth. 2013 Aug 27;13:165. doi: 10.1186/1471-2393-13-165 (PMC3765853; doi:10.1186/1471-2393-13-165)
Supplement: Additional file 3 — Sampling frame used by the studies included in the systematic review. Table detailing the sampling frame used by each study. [file 1471-2393-13-165-S3.doc]

| Study | Pregnancy-based sample | cross-sectional population-based | population-based birth cohort |
| --- | --- | --- | --- |
| Eijsink et al, 2008 | X |  |  |
| Collin et al, 2006 |  | X |  |
| Smith et al, 2006 |  |  | X |
| Murphy et al, 2002 |  | X |  |
| Zdeb et al, 1984 | X |  |  |
| Tollanes et al, 2007 |  |  | X |
| Tower et al, 2000 | X |  |  |
| Huttly et al, 1990 |  | X |  |
| Hemminki, 1987 |  |  | X |
| LaSala et al, 1987 | X |  |  |
| Hemminki et al, 1985 |  |  | X |

**Appendix S3: Sampling frame used by the studies included in the systematic review**
